# Detection of Gene Flow from Sexual to Asexual Lineages in *Thrips tabaci *(Thysanoptera: Thripidae)

**DOI:** 10.1371/journal.pone.0138353

**Published:** 2015-09-16

**Authors:** Xiao-Wei Li, Ping Wang, Jozsef Fail, Anthony M. Shelton

**Affiliations:** 1 Department of Entomology, Cornell University, New York State Agricultural Experiment Station, Geneva, New York, United States of America; 2 Key Laboratory of Plant Protection Resources and Pest Management, Ministry of Education, Northwest A&F University, Yangling, Shaanxi, China; 3 Department of Entomology, Faculty of Horticultural Science, Corvinus University of Budapest, Budapest, Hungary; Plymouth University, UNITED KINGDOM

## Abstract

Populations of *Thrips tabaci* are known to have two sympatric but genetically isolated reproductive modes, arrhenotoky (sexual reproduction) and thelytoky (asexual reproduction). Herein, we report behavioral, ecological and genetic studies to determine whether there is gene flow between arrhenotokous and thelytokous *T*. *tabaci*. We did not detect significant preference by arrhenotokous males to mate with females of a particular reproductive mode, nor did we detect significant behavioral differences between arrhenotokous males mated with arrhenotokous or thelytokous females in their pre-copulation, copulation duration and mating frequency. Productive gene transfer resulting from the mating between the two modes was experimentally confirmed. Gene transfer from arrhenotokous *T*. *tabaci* to thelytokous *T*. *tabaci* was further validated by confirmation of the passage of the arrhenotokous male-originated nuclear gene (histone *H3* gene) allele to the F_2_ generation. These behavioral, ecological and genetic studies confirmed gene transfer from the sexual arrhenotokous mode to the asexual thelytokous mode of *T*. *tabaci* in the laboratory. These results demonstrate that asexual *T*. *tabaci* populations may acquire genetic variability from sexual populations, which could offset the long-term disadvantage of asexual reproduction.

## Introduction

One of the greatest challenges in evolutionary biology is to assess the relative advantages of sexual and asexual reproduction. Sexual reproduction hypothetically has a disadvantage due to the cost of producing males [1]. However, sexual reproduction counterbalances this disadvantage by preventing the accumulation of deleterious mutations and creating new gene combinations that may enhance adaptation [2, 3]. Sexual reproduction is dominant in eukaryotic organisms [4] although asexual populations or parthenogenesis have potential advantages since they do not waste eggs producing males and can establish populations more rapidly [5]. Asexual reproduction lacks the long-term genetic flexibility offered by genetic variation and recombination [1, 6]. These limitations of asexual organisms could explain why asexuality is considered an evolutionary dead-end [1].

Despite the disadvantages of asexual reproduction and the predominance of sexual reproduction, asexual reproduction or parthenogenesis occurs widely in different taxa and many parthenogenetic species appear to be remarkably successful [7, 8]. In some species, sexual and asexual populations even occur sympatrically [9–15]. The contrast between hypothetically limited genetic variation and the frequent occurrence of asexual reproduction indicates there are mechanisms that counterbalance the disadvantages and prevent asexual reproduction from elimination. It has been reported that asexual species may have considerable genetic diversity acquired through various means [7, 16]. Occasional sexual reproduction in generally asexual populations is one mechanism that provides opportunities for genetic material exchanges to occur and maintain genetic variability in asexual populations [17–21]. Additionally, genetic exchange through sexual reproduction in an asexual lineage may hasten the spread of beneficial genes that enhance their rate of adaptation.

Onion thrips, *Thrips tabaci* (Thysanoptera: Thripidae), is an example in which sexual and asexual reproduction occur sympatrically [22–24]. *T*. *tabaci* is a serious global insect pest because of its direct feeding on many agricultural crops and its ability to transmit viruses [25]. Resistance to many insecticides in *T*. *tabaci* populations has resulted in increased crop damage globally [26–29]. *T*. *tabaci* has been divided into three lineages: one tobacco-associated lineage, and two leek-associated lineages [30]. It has been reported that these three lineages differ in their capacity to transmit plant viruses [31–34] and in their host plant preferences [31, 35, 36]. Among the three lineages, the tobacco-associated lineage is more distant from the leek types based on molecular phylogeny [30] and host plant preferences [31].

The two leek lineages are polyphagous and exhibit two reproductive modes: thelytoky and arrhenotoky. Thelytokous females produce 100% of their progeny asexually (all asexually derived individuals are females). Virgin arrhenotokous females also produce 100% asexually derived individuals (all males), but mated arrhenotokous females produce a mixture of sexually (females) and asexually (males) derived individuals [22, 37]. They can co-occur in the field at the same time [22–24]. Previous studies have suggested arrhenotokous and thelytokous *T*. *tabaci* are genetically isolated [23]. Genetic variability statistics based on the mitochondrial cytochrome c oxidase subunit I (*COI*) gene suggested that leek-associated arrhenotokous and thelytokous *T*. *tabaci* lineages might be considered different cryptic species [30]. A more recent study on *T*. *tabaci* nuclear genes using PCR-SSP analysis also suggests genetic isolation of the sexual and asexual leek lineages [23]. Consequently, arrhenotokous and thelytokous *T*. *tabaci* can be distinguished using nucleotide polymorphisms (SNPs) specific to the two lineages [22]. Of the two leek lineages, the thelytokous (asexual) lineage is more common [38]; in addition, genetic variability has been reported among populations within thelytokous lineages [39, 40]. However, there has not been a report on how the thelytokous lineage of *T*. *tabaci* acquires and maintains genetic variability in its populations. In a recent report from a study of field populations of *T*. *tabaci* in Japan, arrhenotokous *T*. *tabaci* individuals were found to have the same *COI* haplotype as that of the common thelytokous *T*. *tabaci* [41]. The finding of arrhenotokous *T*. *tabaci* in the “thelytokous clade” challenged the identification of the two different reproductive modes by maternally inherited *COI* makers [41], but more importantly it prompted the question whether arrhenotoky in the thelytokous population is the result of recent and local gene flow from an arrhenotokous population.

To date, there have not been behavioral studies nor sufficient genetic data available in the literature to assess conclusively the reproductive isolation between these two sympatric lineages. Investigations of gene flow between populations exhibiting these two reproductive modes will provide information to understand the genetic variation in an asexual lineage of *T*. *tabaci*. Such information is important and desirable for effective management of this global pest.

The purpose of this study was to investigate whether mating and gene transfer occur between arrhenotokous and thelytokous *T*. *tabaci*. Using behavioral, ecological and genetic approaches, we asked the following questions: (i) can arrhenotokous males mate with thelytokous females? (ii) If they can mate, are there differences in their mating behaviors and preference of mate selection for a particular reproductive mode? (iii) does mating have fitness effects on thelytokous females and their offspring? (iv) does gene transfer occur when thelytokous females mate with arrhenotokous males?

## Materials and Methods

### Population Maintenance and Insect Rearing

The leek type arrhenotokous and thelytokous populations used in this study were established from *T*. *tabaci* adults of a confirmed reproductive mode. The populations were originally collected in 2011 from cabbage in a research field (GPS coordinates: 42.873621, -77.029556) of Cornell University’s New York State Agricultural Experiment Station, Geneva, New York. The reproductive mode of the field collected individuals was confirmed by observing the sex ratio in virgin progeny (arrhenotokous strain: 100% male offspring; thelytokous strain: 100% female offspring). Thrips larvae were kept individually in microcentrifuge tubes to ensure the emergence of virgin adults. In case of a field collected adult female at least one larva from her progeny was isolated to ensure the emergence of virgin females. Then the progeny of virgin females was used to set up arrhenotokous (allowing mating between the virgin females and the sons of other virgin females) and thelytokous populations. These two populations were maintained on potted onion plants in separate environmental growth chambers at 20 ± 1°C, 60 ± 5% relative humidity (RH), and a photoperiod of 16L: 8D. The reproductive modes of these two colonies did not change since we established them.

### Mating choice of arrhenotokous males towards arrhenotokous and thelytokous females

A 2 to 5-d old arrhenotokous male was confined with 2 virgin females (virginity was confirmed by isolating pupae prior to experiments), 1 thelytokous and 1 arrhenotokous of the same age (2 to 5-d old), in a cap of a 1.7 ml microcentrifuge tube covered with plastic wrap (diam 0.8 cm × height 0.5 cm). Male behavior was monitored under a stereomicroscope (ZEISS, Stemi 2000, Carl Zeiss Microscopy, Jena, Germany) until the male chose one female for mating. The mated and unmated females were captured and kept in 70% ethanol. Their maternal lineage was determined by a diagnostic PCR analysis of the mitochondrial cytochrome c oxidase subunit I (*COI*) gene alleles using primers specific to the arrhenotokous or thelytokous strains (see details in the gene transfer section). The trial was replicated using 32 mating pairs.

### Comparison of mating behaviors between thelytokous and arrhenotokous *T*. *tabaci* females with arrhenotokous males

A single 2-d old adult virgin arrhenotokous or thelytokous female (virginity was confirmed by isolating pupae prior to experiments) was paired with a 2 to 5-d old adult arrhenotokous male under a video recorder (ZC105 Megapixel Camera, Zarbeco, NJ, USA) and their behaviors were recorded at room temperature. The pre-copulation and copulation duration and mating frequency were recorded during 30 min. A total of 24 replicates were used for the arrhenotokous-arrhenotokous and 21 for the thelytokous-arrhenotokous mating experiments.

### Fitness costs of mating behaviors and male companions on thelytokous *T*. *tabaci* females

Newly emerged thelytokous females were confined in 1.7 ml microcentrifuge tubes with onion leaf tissues (6 mm × 6 mm) either individually or paired with an arrhenotokous male at 20 ± 1°C, 60 ± 5% relative humidity (RH), and a photoperiod of 16L: 8D. Leaf tissues were changed at 24-hr intervals and the number of eggs produced by each female was recorded by using the bottom light of a stereomicroscope to view eggs inserted into the leaf tissue. Longevity, lifetime fecundity and survivorship were calculated for each female. In case of a male death before the female, a new male (2–7 d old) was added to replace the dead one so that the female was accompanied by a male during her entire lifetime.

### Effects of mating on life table parameters of the next generation

Mated and virgin thelytokous females (virginity was confirmed by isolating pupae prior to experiments) were left in 1.7 ml microcentrifuge tubes with a single onion leaf tissue for egg collection. Leaf tissues with eggs were kept at the same rearing condition described above and checked every 12 hr for newly emerged larvae. First instars were transferred individually to new tubes with fresh leaf tissues. Egg durations and hatching rates were recorded. The immature stages were checked every 12 hr for development. Survivorship and developmental times at different stages were recorded. When adults emerged, females were contained individually in tubes with leaf tissues. Leaf tissues were changed at 24-hr intervals and the number of eggs produced by each female was recorded. Longevity and lifetime fecundity were calculated for each female.

### Gene transfer from arrhenotokous males to offspring of thelytokous females

Single nucleotide polymorphic sites (SNPs) in the mitochondrial cytochrome c oxidase subunit I (*COI*) gene [36] and the histone *H3* gene were used as molecular markers to track the maternal lineage and nuclear gene transfer between arrhenotokous and thelytokous *T*. *tabaci* strains used in this study. The cytoplasmically inherited mitochondrial markers were used to provide a maternal genealogy of the two specific strains, and nuclear gene markers were used to detect gene transfer between the two strains resulting from sexual reproduction.

The reproductive modes of the two *T*. *tabaci* strains used in this study were first confirmed by observing the sex ratio in virgin progeny (arrhenotokous strain: 100% male offspring; thelytokous strain: 100% female offspring). Based on the *COI* gene fragment sequences from the arrhenotokous and thelytokous strains used in this study (Genbank acc. nos. KJ495742 to KJ295744), three *COI* gene primers were designed to distinguish different strains [36]: generic primer to both strains (5’-TAAACTTCTGGGTGACCAAAAAATCA-3’) and 2 strain specific primers, arrhenotokous strain (haplotype *COI-A*) specific primer (5’-AACAGCTATTCTCCTTCTTTTATCTC-3’) and thelytokous strain (haplotype *COI-T*) specific primer (5’-GAACAGTATATCCACCTTTATCAACG-3’). A PCR product of 161 bp is diagnostic for the arrhenotokous strain and 351 bp for thelytokous strain. The accuracy of this PCR diagnostic detection was confirmed by DNA sequencing of the PCR products from thrips samples.

**Fig 1:**
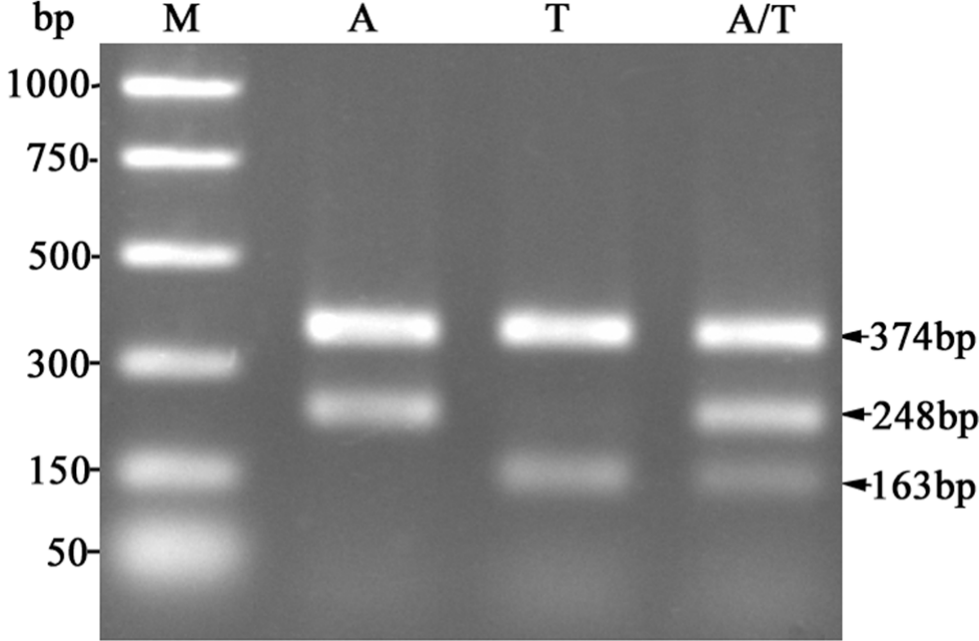
Diagnostic PCR analysis for detection of the arrhenotokous strain and thelytokous strain-originated histone *H3* gene alleles. M: DNA Marker; A: arrhenotokous-originated histone *H3* type; T: thelytokous-originated histone *H3* type; A/T: both arrhenotokous- and thelytokous-originated histone *H3* types.

For the nuclear histone *H3* gene, DNA of a single thrips was extracted as described in our previous work [36]. The histone *H3* gene fragment was amplified by PCR from the arrhenotokous and thelytokous *T*. *tabaci* strains used in this study using primers H3NF: 5’-ATGGCTCGTACCAAGCAGAC-3’ and H3R: 5’-ATATCCTTRGGCATRATRGTGAC-3’ [42]. The PCR products from the thelytokous and arrhenotokous strains were sequenced after a one-step enzymatic purification procedure [43]. From the DNA sequence of the PCR fragments, SNPs in the histone *H3* gene specific to the two strains were identified (Genbank acc. nos. KJ677214 and KJ677215), and two pairs of reproductive mode specific primers were designed: arrhenotokous strain specific primers (H3NF: 5’-ATGGCTCGTACCAAGCAGAC-3’; H3AR: 5’-AAATCGGTTTTGAAGTCTTGC-3’); thelytokous strain specific primers (H3TF: 5’-TTGTTCGAGAAATTGCCCAG-3’; H3R: 5’-ATATCCTTRGGCATRATRGTGAC-3’). Four primers were included in each PCR reaction to genotype the histone *H3* alleles in the samples. The full length of the histone *H3* gene fragment (374bp) was always amplified by primers H3NF and H3R in the PCR reaction, indicative of a successful PCR reaction, regardless of the genotypes in the sample. A 248 bp amplified with the arrhenotokous strain specific primers (H3NF and H3AR) was diagnostic for the arrhenotokous strain-originated allele (*H3-A*), while a 163 bp product from thelytokous strain specific primers (H3TF and H3R) was diagnostic for the thelytokous strain-originated allele (*H3-T*). Consequently, detection of 374bp and 248bp bands indicated the arrhenotokous strain *H3* type; detection of 374bp and 163bp bands indicated the thelytokous strain *H3* type and all the three bands indicated the presence of both arrhenotokous- and thelytokous-strain originated *H3* types (Fig 1).

Twelve pairs of 2 to 4-d old arrhenotokous males (progeny of virgin arrhenotokous females) and thelytokous females (progeny of virgin thelytokous females) were used to test whether thelytokous females used arrhenotokous males’ sperm to fertilize their eggs. The maternal lineage and histone *H3* type of the 12 pairs was confirmed by strain specific diagnostic PCR analysis of the *COI* gene and *H3* gene. Each pair was observed under stereomicroscope until mating occurred. After mating, males were collected and preserved in 70% ethanol for further molecular confirmation of their *COI* and histone *H3* gene alleles. Females were individually transferred into microcentrifuge tubes with cabbage leaf tissue (6 mm × 6 mm) for oviposition. 210 F_1_ offspring (all females) were collected from 12-mated thelytokous females. Virgin F_1_ females (virginity was confirmed by isolating pupae prior to experiments) were allowed to oviposit for 2 wk after which they were examined for their histone H3 genotypes. The *COI* gene marker in F_1_ females was also determined to ensure their lineage from thelytokous mothers. F_2_ offspring from each F_1_ mother were collected and preserved in 70% ethanol and genotyped for *COI* and histone *H3* gene composition, as described above.

### Viability and fertility of F_1_ individuals and the inheritance of the arrhenotokous male-originated gene in the F_2_ generation

The viability and fertility of F_1_ females where gene transfer occurred were examined by analyzing whether they were viable and could produce F_2_ offspring. The *COI* and *H3* genotypes of F_2_ offspring were checked by using the methods described above.

### Statistical Analysis

All data analyses were performed with SPSS software (v16, SPSS Inc., Chicago, IL, USA). Prior to analysis, data were checked for normality using the non-parametric Kolmogorov-Smirnov test (*P* < 0.05) and all percentage data were arcsine transformed, as necessary, but untransformed means are presented. For the data that were not normal (mating frequency, duration of each immature stages), direct estimates were compared using the non-parametric Mann-Whitney *U* test (*P* < 0.05). Data that followed a normal distribution (female pre-copulation and copulation duration, female adult longevity and lifetime fecundity in two generations, duration from egg to adult and survival rates in each immature stage in the F_1_ generation) were compared using Student's *t—*test (*P* < 0.05). For survival analysis of female adults, the Log-Rank test was applied to compare the survival distributions of the female adults between treatments.

Mating choice of arrhenotokous males for arrhenotokous and thelytokous females was analyzed using a Chi-square goodness of fit test (*P* < 0.05). The null hypothesis was that arrhenotokous males showed no mating preference for arrhenotokous or thelytokous females (a selection equal to 1:1).

## Results

### Mating choice of arrhenotokous males towards arrhenotokous and thelytokous females

All 32 males we tested made a choice to mate with one of the females. Arrhenotokous males did not show a statistically significant mating preference for arrhenotokous or thelytokous females (*χ* ^2^ = 2.000, df = 1, *P* = 0.157); of 32 males, 12 chose to mate with arrhenotokous females while 20 chose to mate with thelytokous females.

### Comparison of mating behaviors between thelytokous and arrhenotokous *T*. *tabaci* females with arrhenotokous males

Of 21 thelytokous females tested, 19 mated with males. The mating frequency in 30 min was not significantly different from arrhenotokous females, in which 19 out of 24 mated with males (Table 1; Table A in S1 Data). In addition, no significant differences were found in the pre-copulation and copulation duration (Table 1).

**Table 1:** Comparison of pre-copulation and copulation duration and mating frequency [means ± SE (n)] between arrhenotokous and thelytokous *Thrips tabaci* females with arrhenotokous males.

Reproductive mode	Pre-copulation duration (sec)^*a*^	Copulation duration (sec)^*a*^	Mating frequency in 30 min^*b*^
Arrhenotokous	260.2 ± 88.5 (19)	183.3 ±9.1 (19)	0.96 ± 0.13 (24)
Thelytokous	180.9 ± 29.6 (19)	223.2 ± 26.0 (19)	1.00 ± 0.12 (21)
*t*, df	0.82, 36	-1.45, 36	
*P*	0.419	0.155	0.953

^*a*^ Normal data. Means within the same column were compared by Student's *t* test at *P* < 0.05.

^*b*^ Non-normal data. Means within the same column were compared by Mann-Whitney *U* tests at *P* <0.05.

n: number of replicates used in each treatment.

### Fitness costs of mating behaviors and male companions on thelytokous *T*. *tabaci* females

There were no observed fitness costs to thelytokous *T*. *tabaci* females that mated with arrhenotokous males, nor any significant differences in female longevity and lifetime fecundity between mated and virgin thelytokous *T*. *tabaci* females (Table 2; Table A in S2 Data). In addition, there were no differences in survivorship between mated and virgin thelytokous *T*. *tabaci* (Log-Rank test: *χ* ^2^ = 0.103; df = 1, *P* = 0.748).

**Table 2:** Longevity and lifetime fecundity [means ± SE (n)] of virgin and mated thelytokous *Thrips tabaci* females.

Mating status	Longevity	Lifetime fecundity
Virgin	32.67 ± 2.22 (27)	134.41 ± 10.41 (27)
Mated	30.73 ± 2.82 (26)	135.08 ± 15.11 (26)
*t*, df	0.54, 51	-0.04, 51
*P*	0.591	0.971

Normal data. Means within the same column were compared by Student's *t* test at *P* < 0.05 level. n: number of replications used in each treatment.

### Effects of mating on life table parameters in the next generation

**Fig 2:**
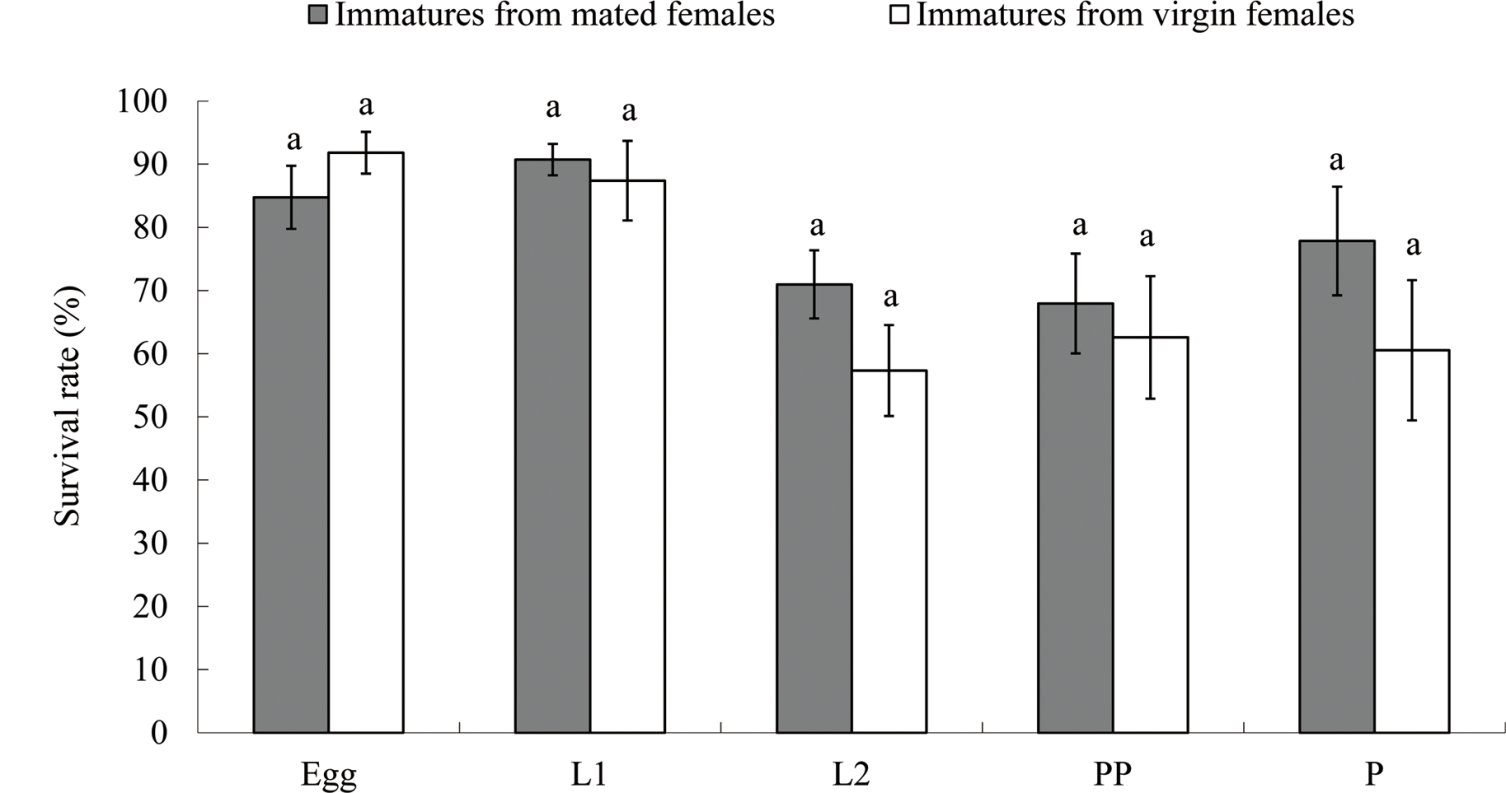
Survival rates of F_1_ immatures from mated and virgin thelytokous female *Thrips tabaci*. **Means within the same stage followed by the same letters are not significantly different at *P* < 0.05 level according to Student’s *t* test.** F_1_: the first generation; L1: first instar; L2: second instar; PP: prepupae; P: pupae.

No differences were observed in the duration of egg, prepupal and pupal stages between offspring from virgin and mated thelytokous females (Table 3; Table B in S2 Data). However, the durations of 1^st^ and 2^nd^ instars, as well as the duration from egg to adult, in offspring from mated females were significantly shorter than those from virgin females (Table 3; Table B in S2 Data). There was no significant difference in survival during all the immature stages between the two treatments (Fig 2; Table C in S2 Data). All F_1_ offspring were female. During the adult stage, the longevity and lifetime fecundity between the two treatments were not significantly different (Table 3; Table D in S2 Data). Furthermore, no differences in survivorship were found between the F_1_ generation of adults from mated and virgin thelytokous *T*. *tabaci* (Log-Rank test: *χ* ^2^ = 0.170; df = 1, *P* = 0.680).

**Table 3:** Life table parameters [means ± SE (n)] of F_1_ offspring ^a^ from mated and virgin thelytokous *Thrips tabaci* females.

Parameters	Offspring from mated females	Offspring from virgin females	
Egg duration (days)^*c*^	6.6 ± 0.06(86)	6.6 ± 0.03(96)	*P* = 0.80
L1 duration (days) ^*c*^	3.0 ± 0.08(77)	3.1 ± 0.06(89)	*P* = 0.01
L2 duration (days) ^*c*^	4.5 ± 0.13(52)	5.7 ± 0.20(55)	*P* < 0.0001
PP duration (days) ^*c*^	1.8 ± 0.07(34)	1.8 ± 0.06(38)	*P* = 0.82
P duration (days) ^*c*^	3.7 ± 0.07(24)	3.8 ± 0.07(28)	*P* = 0.13
Egg to adult (days) ^*b*^	18.7 ± 0.2(24)	20.8 ± 0.3(28)	*t* = -5.01; df = 50; *P* < 0.0001
Longevity (days) ^*b*^	29.0 ± 3.1(24)	29.0 ± 3.3(28)	*t* = 0.03; df = 50; *P* = 0.979
Lifetime fecundity ^*b*^	95.8 ± 15.3(24)	84.9 ± 13.6(28)	*t* = 0.54; df = 50; *P* = 0.539

^*a*^ All the F_1_ offspring were females.

^*b*^ Normal data. Means within the same row were compared by Student's *t* test at *P* < 0.05.

^*c*^ Non-normal data. Means within the same row were compared by Mann-Whitney *U* tests at *P* < 0.05.

n: number of replications used in each treatment; L1: first instar; L2: second instar; PP: prepupae; P: pupae.

### Gene transfer from arrhenotokous males to offspring of thelytokous females

PCR genotyping of the mitochondrial *COI* haplotype of the thelytokous (*COI-T*) and arrhenotokous (*COI-A*) strains and the nuclear gene marker (a fragment of histone *H3* gene) specific to the thelytokous (allele *H3-T*) and arrhenotokous (allele *H3-A*) strains showed that the offspring from mated thelytokous females retained the *COI* haplotype of the thelytokous mother, but the presence of the nuclear histone *H3* gene allele from the arrhenotokous father, allele *H3-A*, was confirmed in some offspring, although at a low frequency. From 12 pairs of crosses tested, the allele *H3-A* was found in the offspring from 4 pairs. Genotyping of the F_1_ individuals from these 4 pairs (all female, 75 individuals in total) determined that 4 of the 75 individuals carried the allele *H3-A*—3 of which were heterozygous (*H3-A*/*H3-T*) and 1 was homozygous *H3-A*. The *COI* gene and *H3* gene haplotype of the 4 individuals were also confirmed by DNA sequencing of the PCR products. The presence of *COI*-*T* and allele *H3-A* in the 4 individuals rules out the possibility of sample contamination and confirmed gene transfer from arrhenotokous males to thelytokous females.

### Viability and fertility of F_1_ individuals and the inheritance of the arrhenotokous male-originated gene in the F_2_ generation

Using 2 of the 4 individuals (a heterozygous *H3-A/H3-T* female and a homozygous *H3*-A female), we tested the viability and fertility of these individuals and the inheritance of the transferred gene. Both individuals were viable and could produce F_2_ offspring. From the homozygous *H3-A* F_1_ individual, all 13 F_2_ offspring tested were *COI-T* for the mitochondrial *COI* gene, demonstrating the maternal lineage of the thelytokous strain, and *H3-A* for the histone *H3* gene, demonstrating the passage of the nuclear gene from the arrhenotokous strain to the F_2_ offspring. From the *H3-A/H3-T* F_1_ individual, all 20 F_2_ offspring tested were *COI-T* for the mitochondrial *COI* gene, and 19 were *H3-A* while 1 was the *H3-T* for the histone *H3* gene.

## Discussion

The arrhenotokous and thelytokous *T*. *tabaci* have been considered to be genetically isolated cryptic species [23, 39]. However, results based on genetic differentiation alone are not sufficient for estimating actual gene flow or hybridization especially when the rate of gene transfer is low [44]. The results from this study demonstrate that mating isolation between arrhenotokous males and thelytokous females is not complete. We did not detect significant behavioral differences between arrhenotokous males mated with arrhenotokous or thelytokous females in their pre-copulation, copulation duration and mating frequency, thus indicating the absence of an observable mating behavioral barrier between the two reproductive modes.

The mating between arrhenotokous male and thelytokous female was mostly not productive. In F_1_ progeny from 12 mating pairs, the arrhenotokous male-originated *H3* gene allele was detected in some individuals from only 4 pairs. In the F_1_ individuals from these 4 pairs, only 4 individuals out of 75 (5.3%) carried the arrhenotokous originated *H3* gene allele. Considering all the F_1_ progeny from the tested 12 pairs, the rate of gene transfer (successful fertilization of eggs of thelytokous females with sperm from arrhenotokous males) was ca. 1.9%. Nonetheless, gene transfer from arrhenotokous male to thelytokous female was clearly demonstrated. The 1.9% successful mating could increase genetic variation in a thelytokous lineage. In addition, gene exchange resulted from mating between reproductive modes could hasten the spread of advantageous trait (i.e. insecticide resistance) in agricultural crops infested by sympatric populations of *T*. *tabaci* and enhance their rate of adaptation.

Gene transfer from sexual males to asexual females has been reported in several other organisms. In the solitary parasitoid wasp *Venturia canescens*, direct crossing experiments in the lab showed that thelytokous wasps are able to mate and receive and use sperm of arrhenotokous males; molecular evidence also documented occurrence of gene transfer from the arrhenotokous to the thelytokous mode in the field [45]. In the water flea *Daphnia magna*, viable mating was reported between females from clones that only produce females, and males from clones that produce both males and sexual females [46]. However, the gene transfer frequency in these organisms was much higher than in *T*. *tabaci*. The higher mating frequency but lower gene transfer frequency suggest there might be gametic isolation involved during the process of fertilization [47]. Nonetheless, low frequency of gene transfer with sexual populations may be sufficient to maintain genetic variation in asexual populations [18], which could promote adaptation in the thelytokous populations of *T*. *tabaci*.

Our study of arrhenotokous and thelytokous *T*. *tabaci* strains sheds light on a recent field study by Sogo et al.[41] that identified arrhenotokous *T*. *tabaci* individuals with the thelytokous mitochondrial *COI* haplotype in field populations in Japan. Arrhenotokous *T*. *tabaci* individuals with different mitochondrial *COI* haplotypes (arrhenotokous or thelytokous type) were identified from populations collected from the same location and also from different locations [41]. These results from field populations of *T*. *tabaci* indicate that some arrhenotokous individuals were produced by females from the thelytokous maternal lineage, which could be achieved by gene transfer from an arrhenotokous male to a thelytokous female by productive mating as observed in our study. Results from our study demonstrated the mating and gene transfer from arrhenotokous *T*. *tabaci* to thelytokous *T*. *tabaci*. However, the extent of gene transfer resulting from the mating between the different modes requires additional work. Nevertheless, the observations reported by Sogo et al.[41] from field populations of *T*. *tabaci* provide evidence that mtDNA from the thelytokous lineage must have introgressed into the sexual or arrhenotokous population in the field, which is consistent with our laboratory confirmation of productive mating and gene transfer from arrhenotokous *T*. *tabaci* to thelytokous *T*. *tabaci*.

The results in this study demonstrated gene transfer from the arrhenotokous lineage to the thelytokous lineage in *T*. *tobaci*. It would be interesting to know whether gene transfer from the thelytokous lineage to the arrhenotokous lineage could also occur in *T*. *tabaci*, as it has been reported that some virgin *T*. *tabaci* could produce both females and males (deuterotokous *T*. *tabaci*) [24]. However, in this study, no males in F_1_ progenies from viable thelytokous females mated with an arrhenotokous male were observed to allow testing potential gene transfer from the arrhenotokous lineage to the thelytokous lineage. The possibility that there could be males produced in the F_2_ generation or even in F_1_ generation at a very low frequency cannot be excluded.

In the present study, of the 4 F_1_ individuals that carried the *H3* gene allele *H3-A*, one was determined to be homozygous *H3-A* and we can confirm the appearance of homozygous *H3-A* is the result of gene transfer by the evidence of coexistence of *COI-T* and allele *H3-A* in this individual and of specific detection of *H3-A* from the diagnostic PCR in the presence of both *H3-A* specific and *H3-T* specific primers. This observation is interesting, but how an F_1_ individual from a cross between an arrhenotokous male and a thelytokous female became homozygous *H3-A* remains unclear. Studies in sciarid flies have shown that elimination of paternally derived whole X chromosomes associated with sex determination occurred in early embryonic cleavage [48]. Also, sex-specific chromatin diminution on an internal portion of one of the two homologs of one chromosome pair has been observed in nematodes [49]. In some pseudo-arrhenotokous arthropods the entire paternal genome is lost during early embryogenesis [50]. Maternal chromosome elimination has also been reported in early embryonic mitotic divisions in *Drosophila melanogaster* and *Sciam ocellaris* [51, 52]. Whether a similar chromosome or entire maternal genome elimination mechanism occurs in *T*. *tabaci*, which would have resulted in the loss of the histone *H3* allele from the mother, is unknown.

Another possible reason for the appearance of homozygous *H3-A* genotype in one of the F_1_ individuals might be polyploidy. In *T*. *tabaci*, both diploidy and polyploidy have been identified both in arrhenotokous and thelytokous females [39, 53]. Arrhenotokous males are reported to be haploid [39]. Polyploidy might result in disorder during gametogenesis, which could lead to the loss of maternal gene. This assumption could also be used to explain another unusual finding in our study—unusual genetic makeup of the F_2_ offspring of the F_1_ heterozygous individual in which 19 carried the grandfather’s allele and 1 carried the grandmother’s allele. However, we did not test the ploidy of our samples. In addition, the cytogenetic mechanisms of gametogenesis in both reproductive modes are unknown. Therefore, a better understanding of the genetics and cytogenetic mechanism of gametogenesis in *T*. *tabaci* will be essential to explain the two unusual observations in this study.

Sexual-asexual gene flow can have important implications for the management of this important pest. Studies have reported the increasing incidence of insecticide resistance in *T*. *tabaci* and plant viruses transmitted by this pest [25]. Sexual-asexual gene flow could lead to the spread of insecticide-resistance alleles and/or virus transmission-associated genes from sexual populations to asexual populations, which might contribute to wide distribution and severe damage by thelytokous populations. Understanding the genetic variation and the occurrence of gene flow in different populations could be helpful in managing insecticide resistance and transmission of plant viruses by this pest.

In conclusion, we did not detect significant preference by arrhenotokous males to mate with females of a particular reproductive mode, nor did we detect significant behavioral differences between arrhenotokous males mated with arrhenotokous or thelytokous females in their pre-copulation, copulation duration and mating frequency. Gene transfer was detected, although at a low rate, when arrhenotokous males were crossed with thelytokous females, and the presence of the transferred gene was confirmed in the F_2_ generation. This study documented that mating and successive gene flow between *T*. *tabaci* lineages of different reproductive modes is possible and this external source of genetic variation could be critical for ecological adaptation and evolution of thelytokous populations of *T*. *tabaci* in agricultural systems.

## Supporting Information

S1 Data Raw data about mating behavior differences between arrhenotokous males mated with arrhenotokous or thelytokous females. (XLSX)

S2 Data Raw data about fitness effects of mating on thelytokous *Thrips tabaci* in two generations. (XLSX)
